# Type of dialysis may influence the prognostic value of myocardial perfusion imaging in end stage renal disease: an observational [^13^N]NH_3_ PET/CT study

**DOI:** 10.1186/s13550-026-01522-9

**Published:** 2026-10-24

**Authors:** Christian Wenning, Thekla Maria Gosmann, Alexis Vrachimis, Michael A. Schäfers, Stefan Reuter

**Affiliations:** 1 Department of Nuclear Medicine, University Hospital Münster, Münster, Germany; 2 Department of Nuclear Medicine, Bonifatius Hospital Lingen, Wilhelmstrasse 13, 49808 Lingen (Ems), Germany; 3 Department of Internal Medicine, Marienhospital Osnabrück, Osnabrück, Germany; 4 Department of Nuclear Medicine, German Medical Center, Limassol, Cyprus; 5 European Institute for Molecular Imaging (EIMI), University of Münster, Münster, Germany; 6 Department of Internal Medicine D, General Internal Medicine and Nephrology, University Hospital Münster, Münster, Germany

**Keywords:** End-stage renal disease, Dialysis, Atherosclerotic plaque burden, Coronary artery calcium score, Myocardial blood flow

## Abstract

**Background:**

Patients with end-stage renal disease (ESRD) exhibit an elevated risk of cardiovascular events, with imaging-based risk stratification becoming increasingly important. This study compares the prognostic utility of [^13^N]NH_3_ PET/CT assessing myocardial blood flow (MBF), coronary flow reserve (CFR) and coronary artery calcium score (CACS) as well as 3D carotid ultrasound (for carotid plaque burden; cPB) in hemodialysis (HD) versus peritoneal dialysis (PD) patients listed for kidney transplantation.

**Results:**

41 ESRD patients (HD *n* = 31; PD *n* = 10; median follow-up 4.9 years) without known coronary artery disease underwent myocardial perfusion imaging with [^13^N]NH_3_ PET/CT, CACS, and 3D carotid ultrasound. HD patients exhibited lower myocardial stress blood flow (MBF_stress_) and CFR compared to PD patients (2.67 ± 0.67 vs. 2.97 ± 0.69; *p* = 0.24 and 2.5 ± 0.74 vs. 3.08 ± 1.12; *p* = 0.07). In the HD subgroup, only MBF_stress_ correlated inversely with CACS and cPB (*r* = -0.32; *p* < 0.05 and *r* = -0.44; *p* < 0.05), whereas CFR did not (*r*=-0.14; *p* = 0.47 and *r*= -0.14; *p* = 0.44). On the other hand, both MBF_stress_ and CFR were predictive of cardiovascular events in HD patients, although the number of events was low (*n* = 4). In contrast, PD patients exhibited only one single cardiovascular event in the follow-up and no significant imaging correlations were observed.

**Conclusions:**

MBF_stress_ and CFR may emerge as prognostic markers for cardiovascular events in HD patients whereas their prognostic power in PD patients remains uncertain. HD patients show more pronounced impairment in MBF_stress_ and atherosclerotic plaque burden in the carotid artery, highlighting particularly this parameter as a prognostic marker in this group.

## Introduction

Cardiovascular disease (CVD) remains the leading cause of morbidity and mortality among patients with end-stage renal disease (ESRD), accounting for over 50% of deaths in dialysis populations [1]. The burden of CVD is disproportionately high in this group due to a complex interplay of traditional risk factors (e.g., hypertension, diabetes, dyslipidaemia) and non-traditional, uraemia-related risk factors such as chronic inflammation, vascular calcification, and left ventricular hypertrophy [2]. 

As renal function declines, patients develop both macrovascular (atherosclerosis, arterial stiffness) and microvascular (capillary rarefaction, endothelial dysfunction) alterations that adversely affect myocardial perfusion and contribute to adverse cardiac events, often in the absence of clinically manifest macroscopic coronary artery disease. As such, early identification of subclinical microvascular impairment is critical, particularly in the context of pre-transplant assessment [3]. 

Kidney transplantation significantly improves long-term outcomes in ESRD patients; however, it is associated with perioperative cardiovascular risk. Consequently, precise cardiac risk stratification is essential prior to listing patients for transplantation. Invasive coronary angiography is avoided in this population due to contrast agent exposure and procedural risks, making non-invasive myocardial perfusion imaging modalities especially valuable [4]. 

Hybrid imaging approaches combining positron emission tomography/computed tomography (PET/CT) and three-dimensional carotid ultrasound allow for comprehensive cardiovascular risk assessment. [^13^N]NH_3_ PET/CT permits absolute quantification of myocardial blood flow (MBF) and coronary flow reserve (CFR), enabling early detection of microvascular dysfunction [5]. Concurrently, coronary artery calcium scoring (CACS) derived from CT imaging provides structural insights into coronary atherosclerosis, while 3D carotid ultrasound quantifies atherosclerotic plaque burden as a surrogate for systemic vascular disease [6–8]. 

While previous studies explored atherosclerosis and myocardial perfusion imaging in ESRD patients, little is known on the prognostic value of these tools, especially with respect to different types of dialysis. Hemodialysis (HD) and peritoneal dialysis (PD) differ significantly in terms of fluid dynamics, hemodynamic stress, inflammatory milieu, and endothelial response—all of which may influence cardiovascular structure and function [9]. 

This study aims to assess the prognostic value of multimodal cardiovascular imaging in a cohort of patients on dialysis without known coronary artery disease. We focussed on comparing HD and PD patients with regards to quantitative myocardial perfusion, atherosclerotic burden, and cardiovascular outcomes, using quantitative [^13^N]NH_3_ PET/CT and 3D carotid ultrasound. Building on our prior cohort report demonstrating stronger associations of MBF_stress_ than CFR with coronary calcification and clinical outcomes [10], we now have tested whether diagnostic and prognostic information differ by the type of dialysis (HD vs. PD).

## Methods

### Study population

This prospective observational study enrolled 41 ESRD patients (13 female; mean age of 51 ± 13 years (range: 26–69), 31 on HD and 10 on PD) referred for kidney transplant evaluation in our center. The cohort consists of the same patient group as in our previous publication [10], supplemented by 2 additional patients and encompassing a longer follow-up period. Exclusion criteria included renal failure in a pre-dialysis state, previous kidney transplantation, or known CAD. All participants were asymptomatic at the time of imaging. Further patient characteristics are detailed in Table 1. All patients underwent hybrid imaging consisting of [^13^N]NH_3_ PET/CT to assess MBF_stress_ and CFR including a low-dose CT for attenuation correction and CACS assessment, and 3D carotid ultrasound for plaque burden quantification (cPB). Cardiovascular events were defined as cardiac death, non-fatal myocardial infarction, unstable angina requiring hospitalization, coronary revascularization, or heart failure hospitalization. Two clinical reviewers adjudicated events by review of medical records during the follow-up time using clinical examinations and patient interviews. Reviewers were blinded to multimodal imaging findings.

**Table 1:** Patients’ characteristics

Variable	Total (*n* = 41)	PD (*n* = 10)	HD (*n* = 31)	*p*-value
Men	28 (68.0%)	5 (50.0%)	23 (74.2%)	0.24
Women	13 (32.0%)	5 (50.0%)	8 (25.8%)	
Age (*years*)	51.1 ± 13.1	50.4 ± 12.7	51.7 ± 13.5	0.8
Time on dialysis (years)	2.6 ± 2.9	1.6 ± 1.7	2.9 ± 3.2	0.19
BMI (*kg/m*^*2*^)	26.8 ± 4.5	26.6 ± 4.3	26.7 ± 4.6	0.92
Atrial fibrillation	1 (2.0%)	0 (0.0%)	1 (3.2%)	1.00
Hypertension	41 (100.0%)	10 (100.0%)	31 (100.0%)	-
Diabetes mellitus	9 (21.0%)	3 (30.0%)	6 (19.4%)	0.66
Hyperlipidemia	24 (56.0%)	7 (70.0%)	15 (48.4%)	0.29
Smoker	21 (49.0%)	3 (30.0%)	18 (58.1%)	0.16
Cardiovascular events	5 (12.0%)	1 (10.0%)	4 (13.0%)	0.56
Death	4 (10.0%)	2 (20.0%)	2 (7.0%)	0.25

*PD*, peritoneal dialysis; *HD*, hemodialysis

### PET acquisition

PET imaging was conducted using a 128-slice PET/CT scanner (Biograph mCT, Siemens Healthcare). Patients fasted for > 4 h and abstained from caffeine or theophylline for 24 h prior to imaging. A non-ECG-gated low-dose CT scan of the heart (100 kV, 60 mA with CARE Dose, 0.5 s per rotation, Pitch 1.2; 3 mm reconstructed slice thickness) was performed during shallow breathing for PET attenuation correction. For the rest study, [^13^N]NH_3_ (357 ± 38 MBq) was administered intravenously. List-mode 3-dimensional PET images were acquired for 14 min. Adenosine infusion (0.14 µg/kg/min, 6 min) was started via a separate intravenous line, with a second dose of [^13^N]NH_3_ (349 ± 39 MBq) given midway through adenosine administration; another 14-minute list-mode acquisition followed. Quality control was applied for both PET and CT attenuation correction. List-mode data were resampled into attenuation-corrected static, electrocardiographically gated, and dynamic images (17 frames: 12 × 10 s, 3 × 20 s, 2 × 30 s). Emission data were adjusted for randoms, dead time, scatter, and attenuation. Images were reconstructed using a 3D attenuation-weighted ordered-subsets expectation maximization algorithm with time-of-flight (OSEM 3D; 2i21s, Gaussian smoothing at 8 mm full-width at half-maximum; zoom 1). PET data analysis was conducted using syngo.PET Myocardial Blood Flow and MI Cardiology software (syngo.via; Siemens Healthineers, Germany).

### Quantification of MBF and CFR

Absolute MBF (ml/g/min) was derived from dynamic stress and rest scans corrected for motion. Automated factor analysis generated arterial input functions and myocardial tissue time-activity curves. Hyperemic MBF (MBF_stress_) was adjusted for myocardial [^13^N]NH_3_ activity (from the rest study). MBF_stress_ and resting MBF (MBF_rest_) were calculated by fitting time-activity curves to a 2-compartment kinetic model as previously described [11, 12]. Global CFR was determined as the ratio of MBF_stress_ to MBF_rest_. Regional analyses were not performed.

### Coronary artery calcium scoring

CACS was assessed from low-dose, non-contrast multidetector CT scans for PET attenuation correction without prospective ECG-gated acquisition. CACS was calculated by the Agatston method, based on the area of coronary artery calcification in CT (> 130 Hounsfield units), and expressed as an Agatston score categorized as ≤ 100; 101–400; 401–1000; and > 1000 [13]. 

### 3D carotid ultrasound

The Philips iU22 system (Philips Healthcare, Bothell, Washington) with a dedicated 3D-transducer was used for all ultrasound scans, which were performed by physicians experienced in head and neck imaging. The protocol included 3D-imaging of the left and right common carotid artery and bifurcation, as far as accessible. Dedicated software (Philips QLAB-VPQ) provided semiautomatic quantification of plaque burden (cPB), reported in mm³ [8]. Patients were stratified into cPB tertiles: ≤ 154 mm³ (low burden), 155–344 mm³ (intermediate burden), and ≥ 345 mm³ (high burden).

### Statistical analysis

Discrete variables were analyzed using the chi-squared test or fisher’s exact test where appropriate. Continuous variables were compared using a two-sided unpaired Student’s t-test or one-way ANOVA with Bonferroni or Tamhane correction, as appropriate. Univariate logistic regression analyses examined associations between continuous variables. Pearson’s correlation coefficient was used to assess relationship between continuous variables. Two-sided p values < 0.05 were considered statistically significant. Kaplan–Meier methods were used to estimate cumulative event-free and overall survival, and differences between groups were evaluated using the LogRank test. Despite low event rates in the cohort cox proportional hazards models were applied to estimate hazard ratios (HR) with 95% confidence intervals (CI). Follow-up time was calculated from the myocardial perfusion imaging date to the first occurrence of the endpoints cardiovascular event or death of any cause. Individuals without an event were censored at the date of last follow-up, loss to follow-up, withdrawal, or administrative study end, whichever occurred first. To assess whether the prognostic association of MBF_stress_ and CFR with overall or event-free survival differed according to dialysis modality, interaction terms between dialysis modality and the respective continuous PET-derived parameter were introduced into Cox proportional hazards regression models with interaction. MBF_stress_ and CFR were centered around their respective sample means prior to calculation of the interaction terms. The models included the respective PET parameter, dialysis modality, and the corresponding interaction term (MBF_stress_ × dialysis modality or CFR × dialysis modality). A statistically significant interaction term was considered indicative of effect modification by dialysis modality.

All tests were two-sided, and p-values < 0.05 were considered statistically significant. All analyses were performed using SPSS Statistics (IBM, Version 30.0, Armonk, NY).

All procedures complied with institutional and research committee ethical standards and the principles of the 1964 Declaration of Helsinki and its amendments. Institutional review board approval was obtained from the Medical Association of Westfalen-Lippe and the Faculty of Medicine, University of Münster (ethics vote file number: 2016-651-f-S). Informed consent was obtained from all patients.

## Results

Baseline characteristics are presented in Table 1. Mean age was 51 ± 13 years in the total cohort without significant differences between men and women or between the dialysis groups (50.4 ± 12.7 years vs. 51.7 ± 13.5 years, *p* = 0.8). The median time on dialysis was 1.5 years in the overall cohort. Although the median time on dialysis was longer in the HD group (1.97 years) than in the PD group (0.91 years), the difference did not reach statistical significance (*p* = 0.09). Median follow-up time was 4.9 years in the overall cohort.

In the total cohort, time of dialysis correlated inversely with MBF_stress_ (*r*= -0.592, *p* < 0.001) and positively with CACS (*r* = 0.37, *p* < 0.05) but not significantly with CFR (*r*= -0.21, *p* = 0.19). When stratified by type of dialysis, these correlations were only observed in HD patients but not in PD patients. LVEF was reduced (< 50%) in 29% in the total cohort, without significant difference between the PD and the HD group (54.4% ± 8.6% vs. 54.3% ± 8.6%; *p* = 0.96).

**Fig. 1:**
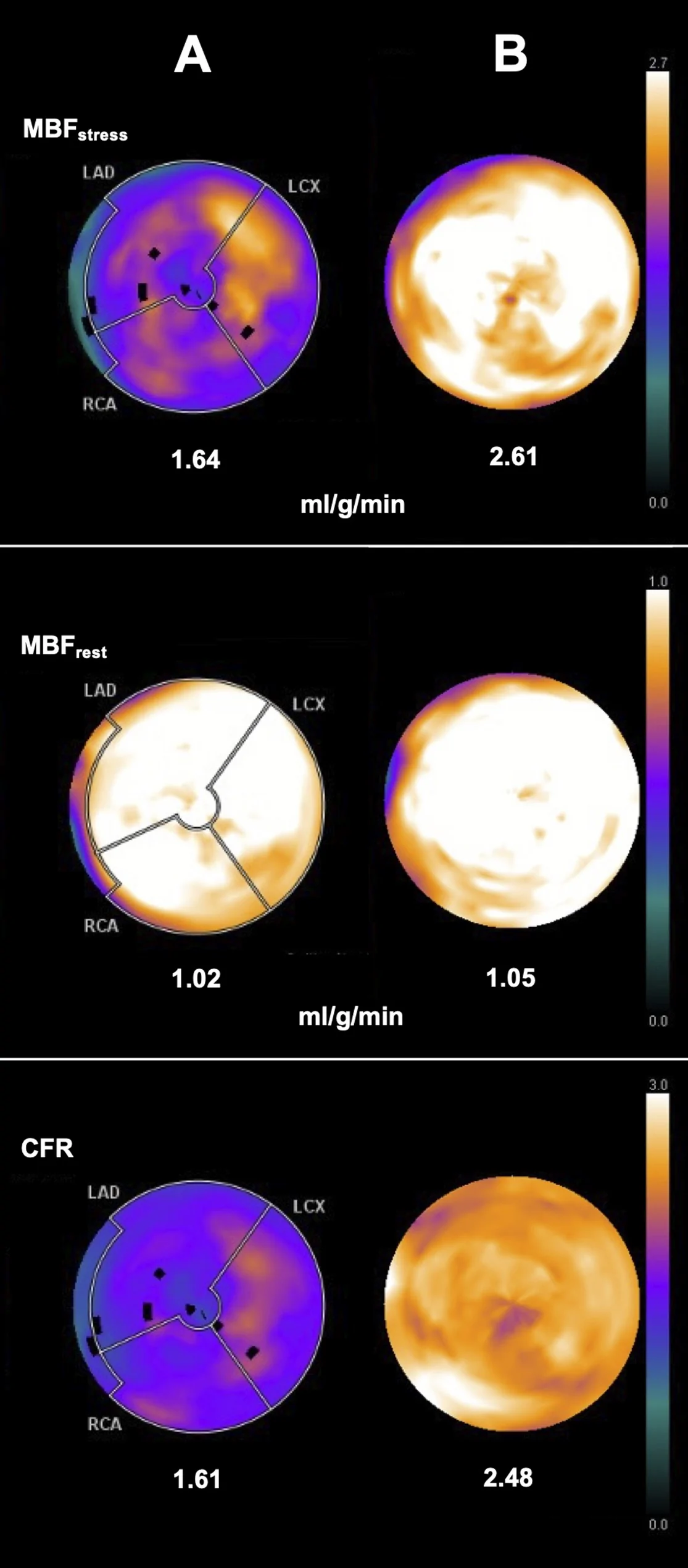
An illustrative comparison between myocardial PET perfusion parameters between a 70-year-old male HD patient (**A**) and a 69-year-old female PD patient (**B**). Both patients had nephrosclerosis leading to ESRD requiring dialysis. Patient A had been on HD for 4 years, whereas patient B had been on PD for 1 year. *MBF*_*stress*_, myocardial blood flow at stress (ml/g/min); *MBFrest*, myocardial blood flow at rest (ml/g/min); CFR, coronary flow reserve

MBF_stress_ was impaired (< 2.0 ml/g/min) in four patients, all of them in the HD group. CFR was impaired (< 2.0) in eleven patients, 1 (9%) in the PD group and 10 (32%) in the HD group (Table 2). Figure 1 shows an exemplary comparison of myocardial PET-derived perfusion parameters in a HD patient versus a PD patient. HD patients exhibited lower MBF_stress_ and CFR compared to PD patients. Similarly, cPB and CACS were higher in HD patients (Table 3). However, these differences did not reach statistical significance.

**Table 2:** Frequency of impaired myocardial blood flow at stress, elevated cPB, CACS and impaired LVEF

	Total *n* (%)	PD *n* (%)	HD *n* (%)	*p*-value
CFR < 2.0	11 (27%)	1 (9%)	10 (32%)	0.24
MBF_stress_ <2.0 ml/g/min	4 (10%)	0 (0%)	4 (13%)	0.56
cPB ≥ 345 mm³	28 (68%)	5 (50%)	23 (74%)	0.24
CACS > 100	16 (39%)	4 (40%)	12 (39%)	1.0
LVEF < 50%	12 (29%)	4 (40%)	8 (26%)	1.0

*CFR*, coronary flow reserve; *MBF*_*stress*_, myocardial blood flow at stress; *cPB*, carotid plaque burden; *CACS*, coronary artery calcium score; *LVEF*, left ventricular ejection fraction; *PD*, peritoneal dialysis; *HD*, hemodialysis

**Table 3:** Imaging parameters by type of dialysis

Parameter	Total (*n* = 41)	PD (*n* = 10)	HD (*n* = 31)	*p*-value
MBF_stress_ (ml/g/min)	2.77 ± 0.68	2.97 ± 0.69	2.67 ± 0.67	0.24
MBF_rest_ (ml/g/min)	1.10 ± 0.27	1.06 ± 0.34	1.11 ± 0.25	0.6
CFR	2.64 ± 0.89	3.08 ± 1.12	2.50 ± 0.74	0.07
CACS	353 ± 740	299 ± 691	393 ± 783	0.72
cPB (mm³)	288 ± 197	194 ± 138	324 ± 203	0.67

Values are mean ± SD

**Fig. 2:**
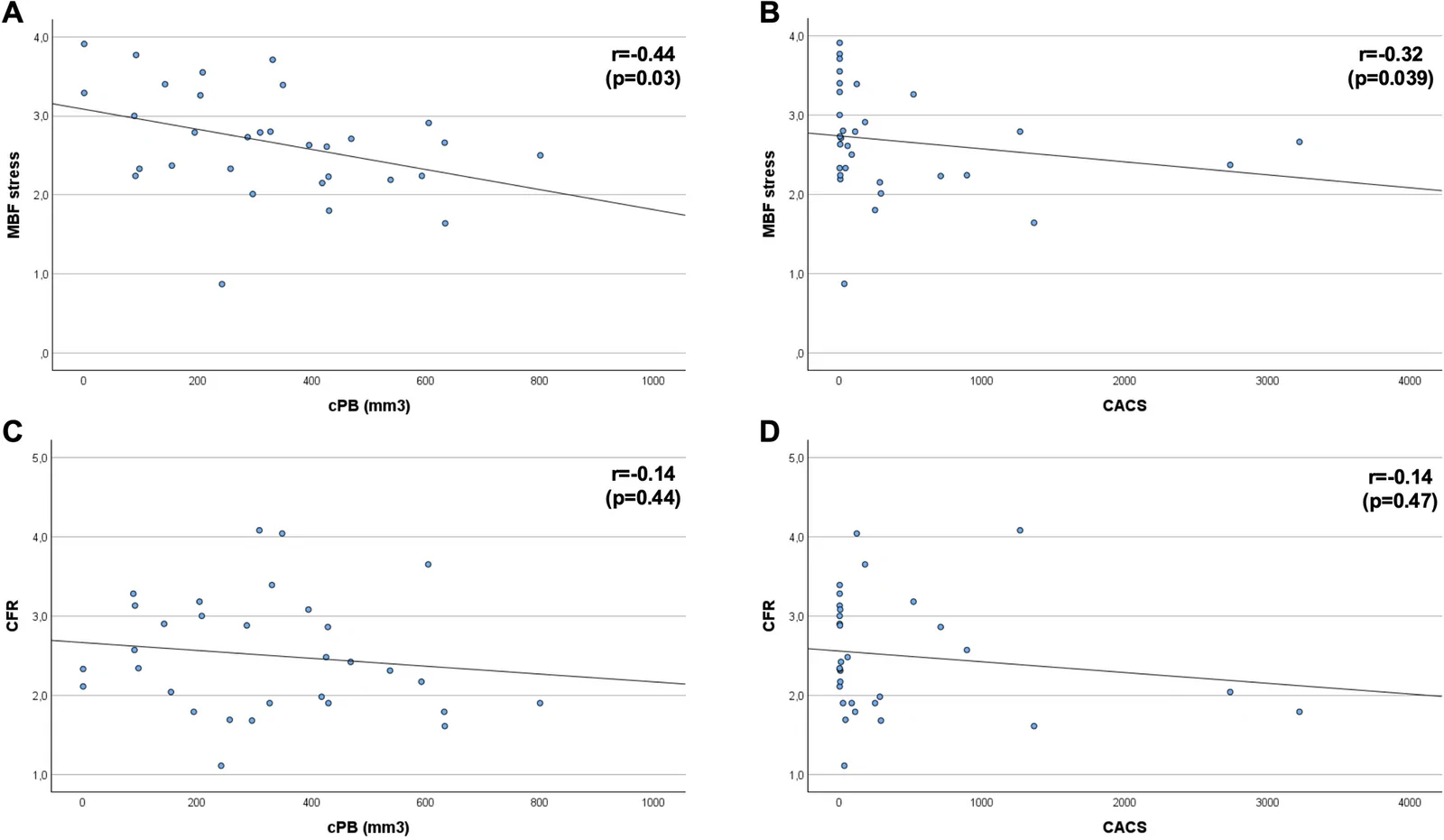
Correlations between MBF_stress_ and CFR with CACS or cPB in HD patients (*n* = 31), respectively. *MBF*_*stress*_, myocardial blood flow at stress; CFR, coronary flow reserve; *CACS*, coronary artery calcium; *cPB*, carotid plaque burden; r=correlation coefficient

In the HD group, MBF_stress_ correlated inversely with CACS (*r* = -0.32; *p* < 0.05) and cPB (*r* = -0.44; *p* < 0.05; Fig. 2A + B), whereas CFR did not (*r*=-0.14; *p* = 0.47 and *r*= -0.14; *p* = 0.44, respectively; Fig. 2C + D). In contrast, in the PD group there were neither significant correlations between MBF_stress_ and CACS (*r* = 0.34; *p* = 0.93) nor between MBF_stress_ and cPB (*r*= -0.03; *p* = 0.8). An unexpected (formally strong) positive correlation between CFR and CACS (*r* = 0.78, *p* = 0.08) was observed in the PD group, likely driven by a single outlier in this small subgroup. No correlation was observed between CFR and cPB (*r*= -0.07; *p* = 0.84).

**Fig. 3:**
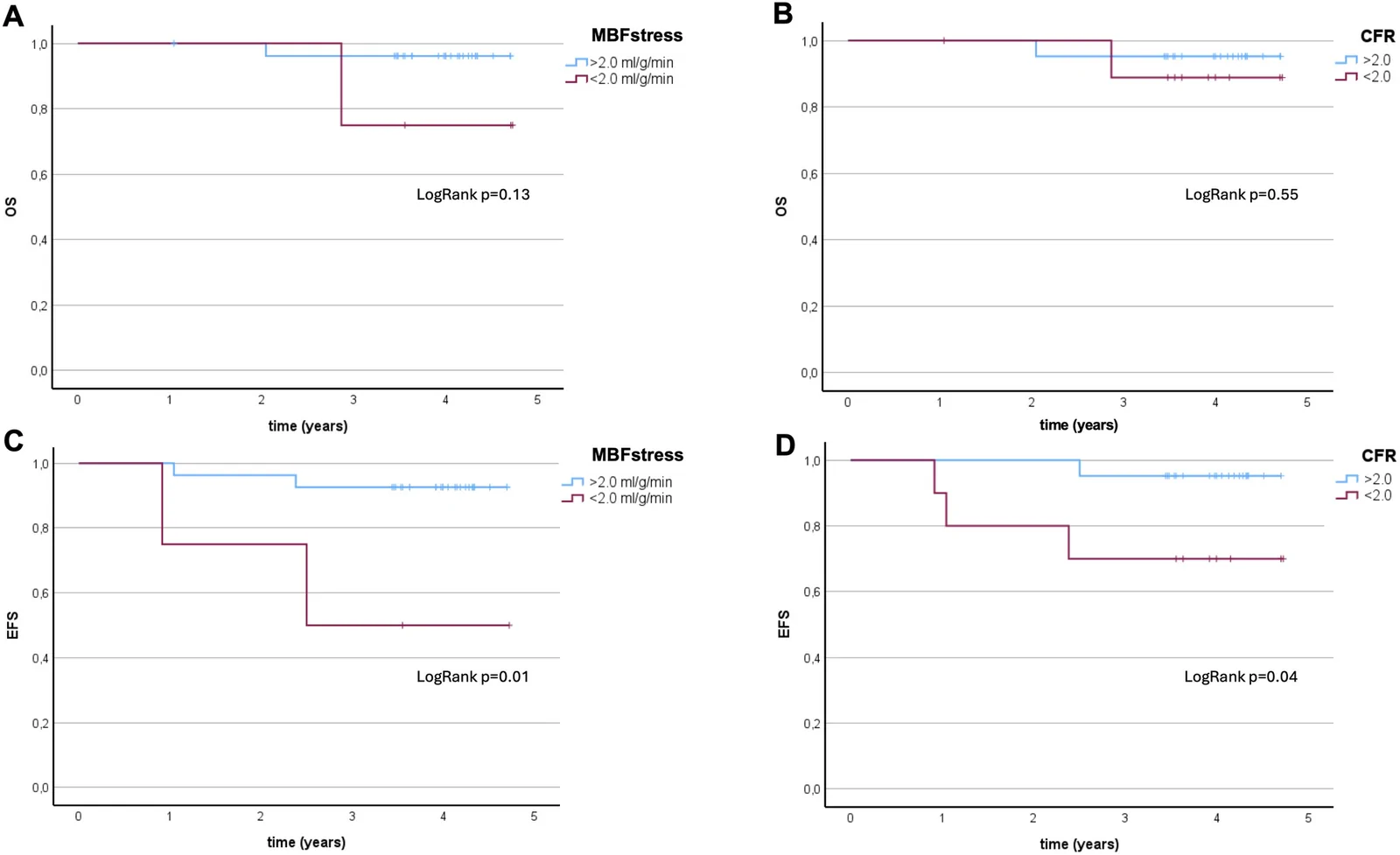
Overall survival (OS; A + B) and event-free survival (EFS; C + D) in HD patients dependent on CFR and MBF_stress_, respectively. Number of events: **A** + **B**: one event in each group **C**: two events in each group **D**: three events in CFR < 2.0, one event in CFR > 2.0. *HD*, hemodialysis; *CFR*, coronary flow reserve; *MBF*_*stress*_, myocardial blood flow at stress

Mean survival time for the entire cohort was 4.5 years (95%CI: 4.18–4.75) without differences between the HD and PD groups (*p* = 0.18). Four patients died during the follow-up period, two patients in each group. Neither MBF_stress_ nor CFR were predictive for death (LogRank p = ns; MBF_stress_: HR 1.94, 95% CI 0.34–11.08, *p* = 0.45; CFR: HR 0.19, 95% CI 0.02–1.98, *p* = 0.17; Fig. 3A + B), and there were no differences between the type of dialysis (not shown).

During follow-up, five cardiovascular events occurred: four (13%) in the HD group and one (10%) in the PD group. Mean event-free survival time was 4.6 years (95% CI: 4.27–4.82). Given the overall low rate of cardiovascular events, in the HD subgroup patients with preserved MBF_stress_ (> 2.0 ml/g/min) or preserved CFR (> 2.0) had longer event-free survival compared to those with impaired MBF_stress_ or impaired CFR (LogRank *p* < 0.05 in each case; MBF_stress_: HR 0.57, 95% CI 0.08–4.14, *p* = 0.58; CFR: HR 0.43, 95% CI 0.4–5.07, *p* = 0.5; Fig. 2C + D). In contrast, MBF_stress_ and CFR were not predictive of events in the PD subgroup, although interpretation is markedly limited by the single event in this group (not shown). Analyses between dialysis modality (HD and PD) and MBF_stress_ or CFR showed no significant interaction, respectively: MBF_stress_: HR 0.16, 95% CI 0.01–2.22, *p* = 0.17; CFR: HR 0.44, 95% CI 0.02–9.71, *p* = 0.61.

## Discussion

In this prospective observational cohort study of ESRD patients undergoing pre-transplant evaluation, our findings suggest that CFR and MBF_stress_ may differ between HD and PD patients and may provide prognostic information for cardiovascular events in HD patients, but not necessarily in PD patients. Patients receiving HD exhibited numerically lower MBF_stress_ and CFR compared with patients treated with PD, although these differences did not reach conventional levels of statistical significance. Additionally, although general patient characteristics and cardiovascular risk factors did not differ between the dialysis groups, atherosclerotic burden was more pronounced in HD patients than in PD patients and CFR and MBF_stress_ were lower, respectively. A limitation to consider in this context is that HD patients had, on average, a longer duration of dialysis than PD patients did. Nevertheless, the strong correlations between MBF_stress_ and CACS and cPB only in HD patients were particularly striking. An additional finding was that both MBF_stress_ and CFR demonstrated prognostic relevance in the HD cohort, whereas no such association can be reliably analyzed among patients undergoing PD due to only one single event in the follow-up and no significant differences between the groups and quantitative PET parameters in interaction analyses. Nonetheless, this observation may suggest that impaired coronary vasodilator function could have clinical significance in HD and could contribute to the elevated cardiovascular risk known to characterize this population. However, these preliminary findings should be interpreted with considerable caution, due to the relatively small sample size and the very low number of recorded cardiovascular events in the follow-up. Event-free survival time was different in the HD group only, tested by Log-Rank test. Non-significant hazard ratios derived from Cox proportional hazard analysis were additionally reported, although their value remains limited due to the low event rate. To clarify in this context, the Kaplan-Meier analyses were based on dichotomized CFR/MBF values, whereas the Cox regression analyses evaluated CFR/MBF as continuous variables.

However, the observed structural and functional abnormalities in HD patients may have contributed to a higher incidence of CVE during follow-up. Consistent with previous observations in the same ESRD cohort, obtained during a shorter follow-up period and without stratification by dialysis modality [10] MBF_stress_ emerged as the more sensitive and robust predictor of adverse cardiovascular outcomes in HD patients compared to CFR. This finding also described in our previous study results [10] could be explained by the fact of increased myocardial resting blood flow in many patients with CKD [14]. In our former study elevated MBF_rest_ correlated with the degree of anemia in ESRD patients but not with imaging markers of atherosclerosis. Further influencing factors could be the commonly observed volume overload in these particular patients leading to increase cardiac workload even at rest due to an enhanced activation of the sympathetic nervous and renin–angiotensin–aldosterone system, which is chronically activated particularly in patients with ESRD and has an impact on resting coronary artery hemodynamics [15]. Therefore, when interpreting CFR, changes in resting MBF resulting from alterations in metabolic demand should be taken into account [16–18]. Overall, the findings of our studies raise the question of whether absolute MBF_stress_ may represent an earlier and more robust marker of coronary artery dysfunction in patients with ESRD than CFR does. Importantly, in this first myocardial perfusion imaging study in this context to differentiate between dialysis modalities, CFR appeared to have prognostic value in HD patients. Noteworthy, neither CACS nor cPB were significantly associated with outcomes in our cohort underscoring the unique prognostic value of CFR and MBF_stress_. However, the differences observed between the two different types of dialysis studied may reflect distinct pathophysiological mechanisms rather than inherent superiority of one mode of dialysis over another. HD patients face a higher cardiovascular risk than those on PD, largely due to more pronounced hemodynamic stress, greater inflammation, and accelerated vascular damage inherent to HD treatment. First, HD triggers greater chronic inflammation and oxidative stress due to repeated exposure of blood to artificial membranes, contributing to endothelial dysfunction and atherosclerosis [19]. Second, HD is characterized by intradialytic hemodynamic instability, myocardial stunning, and repetitive ischemia that may render absolute hyperemic flow particularly informative, whereas PD offers more stable volume and hemodynamics. These pathophysiological differences may at least partly account for the assumed prognostic value of CFR and MBF_stress_ in HD patients [20]. Third, long-term HD patients experience more vascular calcification and arterial stiffness, both strong predictors of cardiovascular events, likely exacerbated by dysregulated mineral metabolism and uremic toxin accumulation [21]. Taking this into account, the longer median time on dialysis in the HD group may have contributed to their more pronounced atherosclerosis development, although this difference in mean times on dialysis was not significant. Nevertheless, cumulative time on dialysis is a recognized risk factor, as prolonged treatment causes chronic low-grade inflammation and oxidative stress, which promote atherosclerosis development and progression [22]. 

The more favorable imaging and clinical profiles observed in PD patients basically align with prior research indicating that PD may be associated with better preservation of cardiovascular function, particularly in the early stages of dialysis [23]. This may be attributed to more stable hemodynamics, less pronounced fluid shifts, and potentially reduced systemic inflammation in comparison to HD [9]. Observational studies, including those from the United States Renal Data System (USRDS) and European Renal Association (ERA-EDTA) registries, have shown variable mortality outcomes between HD and PD, with some suggesting initial survival benefits in PD, especially among younger or non-diabetic patients [24, 25]. A large analysis published reported significantly lower in-hospital mortality for PD versus HD patients undergoing cardiac surgery, along with fewer complications [26]. On the other hand, a Cochrane Review concluded that the risk ratio for cardiovascular death between PD and HD was nearly equal [27]. Moreover, a meta-analysis from 2019 found no significant differences in clinical outcomes between PD and HD [28]. In our study, in the PD group there were more women by trend and time on dialysis was slightly but not significantly shorter than in the HD group. Nonetheless, there were no significant differences between the two groups concerning cardiovascular risk factors or co-morbidities.

The imbalance in group sizes and low event rates limits direct comparisons between dialysis modalities and precludes robust conclusions regarding differential prognostic effects. Given these constraints, the current findings should be regarded as an exploration and hypothesis-generating rather than definitive evidence of modality-specific differences in coronary microvascular dysfunction or cardiovascular risk stratification. Nevertheless, the study provides preliminary indications that quantitative PET-derived perfusion parameters may capture clinically relevant alterations in coronary vascular function among patients undergoing evaluation for kidney transplantation. Future multicenter studies with larger cohorts and a greater number of cardiovascular events are warranted to validate these observations and to determine whether modality-specific differences in myocardial perfusion translate into meaningful differences in clinical outcomes.

In particular, the small number of PD patients analyzed (*n* = 10) precludes meaningful conclusions regarding a potential prognostic impact. Even so, observations in this small group showed that MBF_stress_ and CFR values were higher than those in the HD group were and only one cardiovascular event occurred during the follow-up. However, this single event in the small PD subgroup precludes meaningful statistical comparison of event rates or prognostic markers between modalities. Despite the small sample size, the absence of significant correlations between perfusion and atherosclerosis markers in this group could suggest that PD may exert a less deleterious effect on cardiovascular structure and function, although the limitations in cohort size preclude our results from providing conclusive evidence for this hypothesis. However, when interpreting cardiovascular imaging in ESRD standardized risk assessment protocols incorporating modality-specific imaging findings could improve pre-transplant decision-making and long-term outcomes. The integration of CFR and MBF_stress_ assessment into clinical workflows may help to identify high-risk HD patients who could benefit from intensified surveillance or preventive interventions. The study cohort represents a highly selected population, comprising individuals without known CAD but with markedly elevated cardiovascular risk. Therefore, our findings are only applicable only to this small and highly specific patient cohort and should not be generalized to the broader population.

### Limitations

Several important limitations substantially restrict the strength and generalizability of the present results. First, the study cohort was relatively small, particularly in the peritoneal dialysis group (*n* = 10), resulting in limited statistical power to detect potentially meaningful differences between treatment modalities. Second, the number of cardiovascular events observed was very low (four events in the HD and one event in the PD group). As a result, survival analyses were based on a small number of outcome events, increasing the risk of unstable estimates and chance findings. The apparent prognostic value of myocardial blood flow and coronary flow reserve in the hemodialysis cohort therefore requires confirmation in larger prospective studies.

Particularly the small PD subgroup with only one cardiovascular event remains a major limitation of this observational study with markedly limit statistical power and precision. The absence of detectable associations in the PD group should not be interpreted as evidence of no effect, but rather as insufficient data for meaningful analysis. Residual confounding (e.g., nutrition, dialysate glucose, membrane biocompatibility) cannot be excluded. The single-center design of the study suggests that the generalizability of the results is limited. The adjudication of events was not centralized; however, prespecified criteria were employed.

## Conclusion

The results of this hypothesis-generating study indicate that [^13^N]NH_3_ PET/CT-derived myocardial perfusion parameters MBF_stress_ and CFR may have prognostic value in predicting cardiovascular events in HD patients, whereas our data do not allow for a reliable assessment of their prognostic value in patients with PD. These preliminary findings suggest that the interpretation of myocardial perfusion imaging may need to account for dialysis modality. Given the small patient cohort and the limited number of events in the follow-up, the statistical power of the study is substantially limited. Thus, larger studies are needed to validate these observations in these pre-transplant CKD patients and determine whether dialysis-specific risk stratification strategies improve pre-transplant evaluation.

## Data Availability

The datasets used and/or analyzed during the current study are available from the corresponding author on reasonable requests. Original data may be made available on reasonable requests.

## Abbreviations

BMI: Body mass index
CACS: Coronary artery calcium score
CAD: Coronary artery disease
CI: Confidence interval
CFR: Coronary flow reserve
cPB: Carotid plaque burden
CVD: Cardiovascular disease
CVE: Cardiovascular events
ECG: Electrocardigram
ESRD: End-stage renal disease
HD: Hemodialysis
HR: Hazard ratio
LVEF: Left ventricular ejection fraction
MBF: Myocardial blood flow
MBF_stress_: Myocardial blood flow at stress
MBF_rest_: Myocardial blood flow at rest
MI: Myocardial imaging
PD: Peritoneal dialysis
PET/CT: Positron emission computed tomography/computed tomography
